# Evaluation of Hybrid Melamine and Steel Fiber Reinforced Geopolymers Composites

**DOI:** 10.3390/ma13235548

**Published:** 2020-12-05

**Authors:** Patrycja Bazan, Barbara Kozub, Michał Łach, Kinga Korniejenko

**Affiliations:** Institute of Material Engineering, Faculty of Material Engineering and Physics, Cracow University of Technology, Jana Pawła II 37, 31-864 Cracow, Poland; barbara.kozub@pk.edu.pl (B.K.); michal.lach@pk.edu.pl (M.Ł.)

**Keywords:** geopolymer, fly ash based geopolymer, steel fiber, melamine fiber, hybrid reinforcement

## Abstract

This study investigated the influence of the steel and melamine fibers hybridization on the flexural and compressive strength of a fly ash-based geopolymer. The applied reinforcement reduced the geopolymer brittleness. Currently, there are several types of polymer fibers available on the market. However, the authors did not come across information on the use of melamine fibers in geopolymer composites. Two systems of reinforcement for the composites were investigated in this work. Reinforcement with a single type of fiber and a hybrid system, i.e., two types of fibers. Both systems strengthened the base material. The research results showed the addition of melamine fibers as well as steel fibers increased the compressive and flexural strength in comparison to the plain matrix. In the case of a hybrid system, the achieved results showed a synergistic effect of the introduced fibers, which provided better strength results in relation to composites reinforced with a single type of fiber in the same amount by weight.

## 1. Introduction

Geopolymers are inorganic polymers, formed from substances rich in silicon (Si) and aluminum (Al) as a result of a polycondensation reaction in a strongly alkaline or acidic environment. They are characterized by very good mechanical and physical properties, including high compressive strength, thermal stability, low shrinkage, as well as resistance to fire and acids. Geopolymers are synthesized at temperatures below 120 °C, which, compared to traditional ceramic materials, results in low energy consumption during the production process, and allows to reduce the negative impact on the natural environment. Geopolymers are often used in a construction industry, but they also find important applications in the immobilization of toxic waste and heavy metals, and also for the refractory coatings in aviation equipment. Geopolymers are suitable for the production of geopolymer cements, mortars, and concrete with similar or even better properties than traditional materials used in construction [1,2].

The study of the geopolymer properties showed its excellent compressive but quite poor tensile strength, including brittle behavior. In traditional building materials such as a concrete, short fibers randomly placed with a concrete mixture known as fiber reinforced concrete (FRC) are used to decrease the brittleness of the material. In the case of a material without fibre reinforcement, fracture under the load spreads quickly and can causing a loss of the load-bearing capacity, while the use of fibers as a reinforcement causes capture by fibers, the crack, thereby slowing down its further propagation or even stops it. This effect is called the crack masking effect, thanks to which the hardness of the concrete increases and the material retains the load-bearing capacity despite the formation of the first crack [3]. Due to the fact that the properties of the geopolymers are quite similar to those of the hardened cement, a lot of researches have been done on the development of geopolymer composites reinforced with both natural and synthetic fibers. Table 1 presents examples of applied fibers and the aim of the addition.

Correia et al. investigated the mechanical properties of the geopolymer composites reinforced with natural fibers. Results for the fibers used in the research, included sisal fibers and pineapple leaves, proved that the addition of natural fibers increased the compressive strength and toughness in comparison with the unreinforced metakaolin-based geopolymer. A greater effect was obtained with the use of sisal fibers than with pineapple leaves. However, each of the additives promoted a positive effect on the mechanical behavior [22].

Korniejenko et al. conducted research on geopolymers based on fly ash reinforced with cotton, sisal, raffia, and coconut fibers. Researches pointed that the addition of natural fibers in the form of coconut increased the compressive strength by about 25% and cotton by about 15%. The introduction of raffia fibers caused a decreased in strength properties both in compression and bending. The flexural behavior for the remaining examined composites was comparable to the unreinforced material [23]. Also, some investigation to reinforcement from waste materials, such as coffee grounds were provided [24].

Alternative examples of fibers applied in geopolymers are synthetic fibers, such as aramid, polyacrylinite, polyamide, polyethylene, polypropylene, polyvinyl or polyvinyl chloride fibers [25,26]. Fly ash-based geopolymer composites reinforced with aramid fiber exhibited a rise in compressive strength by about 25% and a strengthen in flexural strength by almost 50% compared to unreinforced geopolymer composites [27]. Research on geopolymer composites with polyamide fibers was also performed. The influence of fiber compactness on the changes of mechanical properties of composites was assessed. The base of the geopolymer was metakaolin. The polyamide fibers were introduced at 0.4%, 0.8%, and 1.2% by volume. The tests were carried out after three, seven, and 28 days of sample production. A positive effect of the polyamide fibers addition on the mechanical properties was found. The investigated properties were increased with rising amount of fiber addition, as well as with extended conditioning time after which the materials were tested [28].

Other examples of synthetic fibers are polyethylene (PE) and polyvinyl alcohol (PVA) fibers. Geopolymer composites based on fly ash and slag were produced with PE and PVA fibers in an amount of 2% by volume. The mechanical properties of those composites were tested after 28 days of conditioning. The test results demonstrated a deteriorate in the compressive strength of the geopolymer with polyethylene fibers and a slight increment of it for the material modified with PVA fibers. The compressive strength was 48.6 MPa for the unreinforced geopolymer, 44.3 MPa for the composite with PE fibers and 48.7 MPa for the composite modified with PVA fibers [29,30]. Properties of geopolymer composites of polypropylene fiber-modified fly ash in volumes of 2%, 3%, 4%, and 5% of fibers were investigated by Ranjbar et al. Experiments revealed that the addition of polypropylene fibers increased the bending strength and decreased the compressive strength. The best results were obtained for the addition of 4% and 5% of fibers [31,32]. Steel fibers also served as reinforcement for geopolymer composites. The research revealed that the addition of steel fibers in the amount of 1% by volume increases the tensile, bending, and compressive strength. The significant influence of the fibers geometry on changes in the properties of the produced geopolymer composites was also demonstrated [33,34,35].

Another type of reinforcement in composites is hybrid reinforcement containing at least two different reinforcements. Such application aims to use various properties of different types of fibers [36,37]. The most common hybrid combination is the connection of the steel and polymer fibers, e.g., the usage of steel fibers and polypropylene fibers in geopolymeric composites based on fly ash and silica fume. The fiber content was constant and equal 1% by volume. However, the ratio of steel to polypropylene fibers was variable. The research proved that the mechanical properties increased with the increase in the amount of steel fibers [3,38]. Another example of connection with the different fibers is combining steel fibers with polyethylene fibers. The research was carried out on two types of steel fibers, spiral and curved. The fibers were introduced into a geopolymer matrix composed of fly ash and slag. The analysis pointed a decrease in compressive strength of samples with hybrid reinforcement compared to materials with the same steel fiber content, the values were similar to the geopolymers matrix gained. On the other hand, hybrid fibers developed the bending properties, and a positive effect of crack propagation inhibition was noticed, confirming the change of cracking character from brittle to more plastic [39,40,41,42].

Man-made fibers are currently the most commonly used addition to various types of composites. The addition of man-made fibers, such as melamine, is primarily aimed at improving mechanical properties, in particular bending strength, and additionally reduction of the propagation of microcracks in the material. Other expected benefits, depending on the type of fiber used, may be, for example, an increase in fire resistance or a decrease in the thermal conductivity coefficient, or other features desirable for a given application. It should be noted that chemical fibers usually have higher strength properties and higher repeatability than natural fibers. In the field of fibers produced from inorganic raw materials, research mainly concerned steel, glass, and carbon fibers. Melamine is considered to be one of the best acoustic and thermal insulation materials available on the market for high temperature applications [43]. The addition of steel fibers allows not only to increase the mechanical properties of composites, but also ensures good coherence of the geopolymer matrix with the filler and reduces corrosion in such composites [44].

This article presents the results of research constituting an attempt to obtain a geopolymer with the addition of melamine fibers, additionally reinforced with steel fibers. The article presents a strong aspect of novelty, because the melamine fibers have been not investigated and applied as a reinforcement for geopolymer composites. Similarly, the hybrid composites with melamine and steel fibers have been not researched yet. The authors examined the effect of the melamine and steel fibers addition on selected properties of fly ash matrix geopolymers, delivered from the bituminous coal power plant ’Skawina’ in Poland. The scope of the tests included: microstructure investigation, density, compressive strength test and bending tests. For manufactured composites based on geopolymer reinforced with melamine and steel fibers, an attempt was made to determine the possibilities of their use as structural materials, in particular as materials for use in civil engineering.

## 2. Materials and Methods

### 2.1. Materials

**Figure 1:**
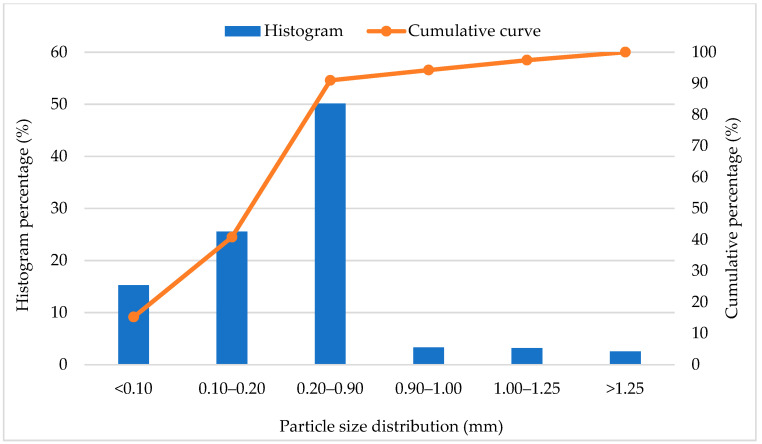
Histogram of particle size distribution and cumulative particle size distribution curve for the construction sand used in the test.

Geopolymers were prepared using the sodium promoter, fine construction sand (saturated-surface dry which means that there was little moisture inside the sand particles) and fly ash acting as a precursor (Skawina Heat and Power Plant, Skawina, Poland). Figure 1 presents the histogram of the particle size distribution and the cumulative particle size distribution curve for the construction sand used in the test (results from own research).

The activation process was carried out with a 10-molar (10 M) solution of sodium hydroxide NaOH in combination with a solution of sodium silicate (water glass) in the ratio of 1:2, which is the most commonly used hydroxide activator in the synthesis of geopolymers.

The use of NaOH as an activator in the synthesis of geopolymers, both from fly ash and other precursors, for example metakaolin, is very widespread due to its low cost, wide availability, and low viscosity. However, the highly corrosive nature of concentrated NaOH or other alkali hydroxide means that very specialized processing equipment would be required to produce large amounts of hydroxide-activated geopolymers [24].

**Figure 2:**
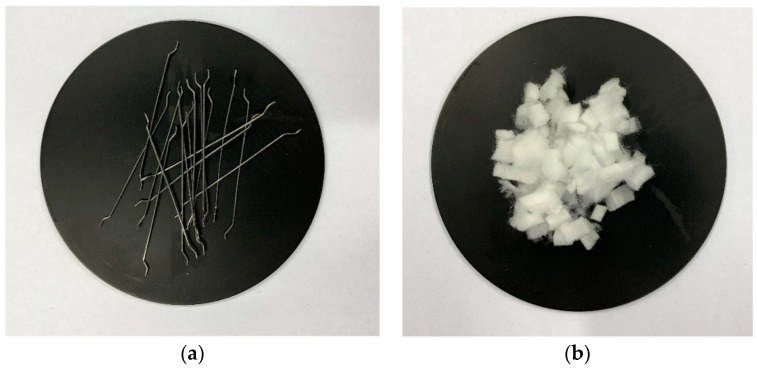
Fibers: 2 (**a**) steel, (**b**) melamine.

Technical sodium hydroxide in the form of flakes and an aqueous solution of sodium silicate R-145 with a module of 2.5 molar and a density of about 1.45 g/cm^3^ were used for the production of geopolymer masses. Tap water was used instead of distilled water. The alkaline solution was prepared by addition to the solid sodium hydroxide the aqueous sodium silicate solution and the water. The solution was mixed thoroughly and allowed to equilibrate to ambient temperature. The following materials were used to prepare the geopolymer mass: 50% fly ash, 50% fine construction sand, 1200 mL of 10 M sodium hydroxide solution together with an aqueous sodium silicate solution. Steel fibers (EKOMET, Ozorków, Poland) and melamine fibers (smartMELAMINE^®^, The smart polymer GmbH, Rudolstadt, Germany) were used to reinforce the composites (Figure 2).

Table 2 shows the characteristics of the fibers.

The solid components of the mixture, i.e., fly ash with the addition of sand and the reinforcement were dry mixed until a homogenous mixture was obtained. The alkaline solution was then added and mixed thoroughly. After obtaining a homogeneous mass with a densely plastic consistency, the mixtures were transferred to molds and subjected to vibrations on a vibrating table. The formed geopolymer concretes were heated in a laboratory dryer (SLW 750 STD, POL-EKO-APARATURA, Wodzisław Śląski, Poland) for 24 h at a temperature of 75 °C under atmospheric pressure. After 24 h, the samples were removed from the molds. Table 3 provides a description of the produced geopolymer composites.

### 2.2. Methods

X-ray diffraction (XRD) (PANalytical, Almelo, The Netherlands) was used to analyze the mineral composition of the used precursor. The phase composition was determined using the powder X-ray method (Debye—Scherrer). Phase analysis was performed on the PANalytical AERIS X-ray diffractometer using Cu-Kα radiation. The parameters and components used during X-ray examinations included: angular range was set from 9999 to 100° 2 Ѳ; measuring step was equal 0.0027166° 2 Ѳ; nickel filter was applied on the lamp, and the knife was in low position. The obtained values of the interplanar distances were used to identify the phases contained in the fly ash. X-ray analysis was performed using the HighScore Plus software. In order to analyze the obtained diffractograms in terms of the presence of phases and to quantify these phases, the PDF4 + crystallographic database was used.

Microscopic observations and energy dispersive spectroscopy (EDS) were carried out on a scanning electron microscope Joel JSM-820 using the EDS (IXRF, Inc., Austin, TX, USA) X-ray detector.

Before the strength tests, the density of the samples was measured by the geometric method. Then the compression test was performed in accordance with EN 12390-3 [45], using Matest 3000 kN testing machine (Matest, Treviolo, Italy), on cubic samples 50 × 50 × 50 mm^3^, conditioned at room temperature for 28 days. The bending strength tests were performed in accordance with the EN 12390-5 standard [46], also on the universal testing machine Matest 3000 kN. The tests were carried out on prismatic samples with dimensions of 50 × 50 × 200 mm^3^ (the distance between the support points was 150 mm). The test speed was 0.05 MPa/s. The obtained result is the arithmetic average of four measurements.

## 3. Results and Discussion

### 3.1. EDS and XRD Studies of the Precursor

The selection of an appropriate precursor for the production of geopolymers depends on many factors. The morphology of the particles is extremely important. It should be regular with relatively spherical shapes because that influences on the rheological properties of the mixture, changes its workability, and also reduces the addition of liquid substances. Additionally, a high content of silica and aluminum oxide is also profitable. The amount of unburned carbon, sulfur compounds, and iron compounds, as well as the content of calcium oxide are also curtailed due to the fact that they are very often considered impurities [47].

**Figure 3:**
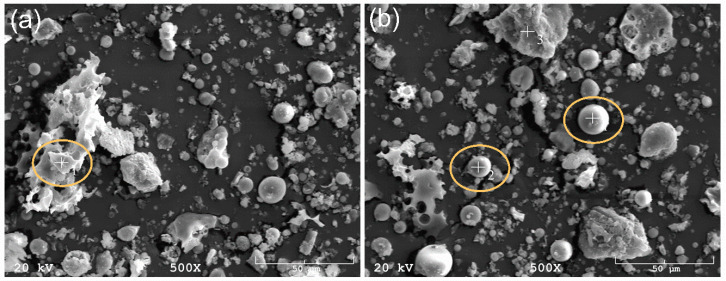
Micrographs of fly ash: point 1 on 3 (**a**) unburned carbon particles, point 1 –2 on 3 (**b**) fly ash with characteristic globular shapes.

The first stage of the research was the assessment of the chemical composition of fly ash. Tests were conducted using energy dispersion spectrometry (EDS), as well as qualitative and quantitative analysis of the phases present in the rasterized precursor using X-ray diffraction (XRD). Fly ash obtained from the ’Skawina’ CHP plant, shown in Figure 3, is characterized by globular particles up to 20 μm (point 1–2, Figure 3b), and particles of unburned carbon with very complex shapes were also observed (point 1, Figure 3a).

Spectrometric analysisshowed that the fly ash contains compounds of silicon, aluminum, calcium and a small amount of iron, magnesium, sodium and titanium, which from the geopolymerization point of view may have a beneficial effect on the mechanical properties of geopolymers.

The titanium present in the ashes can affect the formability of the material, it is present as an impurity in kaolin and other clays. It is known from scientific research that titanium is a good nucleating agent for crystallization. Other types of particles may also be present in the coal fly ash, including reactive calcium silicate phases similar to those found in Portland cement. These particles are the result of cements that fill the pores in the carbon. The presence of calcium in large amounts may interfere with the polymerization process and change the microstructure [24].

The fly ash components are mainly oxides: SiO_2_, Al_2_O_3_, Fe_2_O_3_, CaO, MgO, Na_2_O, K_2_O and TiO_2_. The fly ash also contains trace amounts of such elements as: Ba, Cu, Sr, Ni, Cr, Zn, Cd, Mo, V, Se, Pb, As and others. The loss on ignition is about 2.84% [46]. The properties of fly ash are determined by many factors, with the most important being: the type of coal burned; type of installation in which coal combustion takes place, i.e., type of boiler and technological combustion conditions; fuel preparation method; method of ash capture, discharge and storage; gas desulphurization technology and the type of SO_2_ sorbent used. Each coal particle may contain different amounts of different inorganic substances and thus the resulting ash may be highly heterogeneous [47,48].

XRD analysis displayed that fly ash is characterized by a high content of quartz and mullite in the amount of 42.3 and 54.8%, respectively (Table 4). Some studies on geopolymer materials indicate that a small amount of quartz may have a positive effect on the mechanical properties, and other minerals may have a detrimental effect on the geopolymer [49]. The presence of hematite, magnetite, anhydrite, and rutile was also observed. However, the content of these phases did not exceed 1.5%. Large amount of iron (on the order of a few percent) in the form of hematite or magnetite may adversely affect the disintegration of the ash grains. These compounds are usually formed on the surface of the ash grains and hinder the access of the liquid phase to its vitreous substance. The high content of unburned carbon increases the water demand of the ash. The summary of identified phases is presented in Table 4.

### 3.2. Research on the Mechanical Properties of Geopolymer Composites

**Figure 4:**
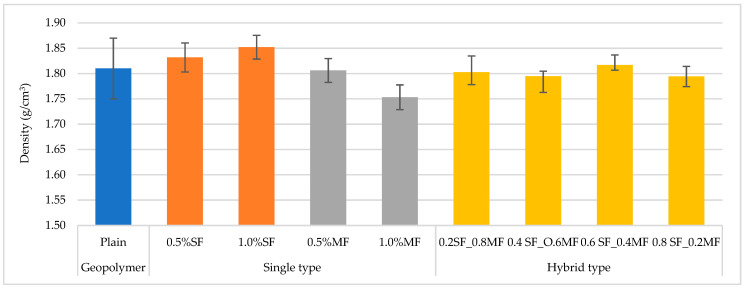
Density results of tested materials.

In the second stage of the research, geopolymer composites based on fly ash and fine construction sand in a 1:1 ratio were produced. Composites were filled with individual types of fibers. As additives steel fibers and melamine fibers were introduced. The fiber content was 0.5% and 1.0% by weight. The results of the density measurement are summarized in Figure 4.

The investigations indicated the density of the materials increased with steel fibers addition. Analyzing the material modified with melamine fiber, the density of the composites is comparable with the base material or slightly lower. The density of melamine fibers is about 1.6 g/cm^3^. However, the fibers are quite highly absorbent, hence a slight increase in the density of the composite, especially when the high value of the measurement error is taking into account nonetheless, the higher the content of melamine fibers, the lower the density of the composite, which is related to the greater weight content of the polymer fibers.

The density of geopolymer composites can vary greatly due to many factors affect it. Polymer fibers have a lower density than geopolymers, while steel fibers have a density comparable or higher. However, using fibers with a higher density than geopolymers not guarantee that the density always increases, and with polymer fibers decrease. This means that the density of the composite can be (1) increased, (2) decreased, or (3) the same as in the case of unreinforced geopolymer fibers due to entanglement processes [50].

**Figure 5:**
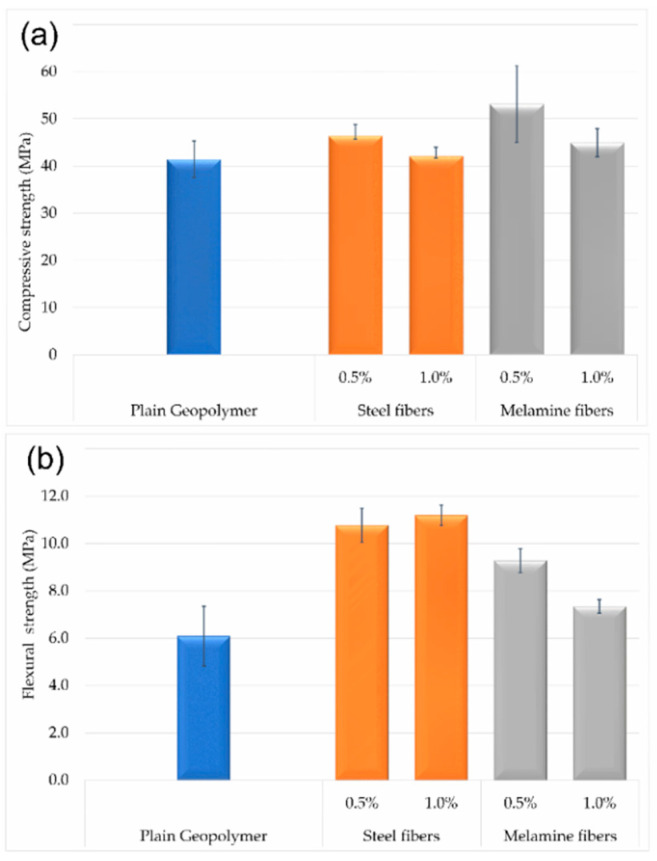
Compressive 5 (**a**) and flexural 5 (**b**) strength of single type reinforcement composites.

Figure 5a,b illustrates the test results obtained from the compression and flexural tests. The investigations of the mechanical properties showed that both the compressive strength and bending strength raised in relation to the base material.

Comparison of the compression properties exhibited a slightly different trend. First of all, it was noticed that the best results were obtained when the additives did not exceed the amount of 0.5% by weight. Further increase in fiber content of both melamine and steel promoted a decrease in compressive strength almost to the level achieved for the unmodified material. Compressive behavior of the composites with melamine fibers presented better resistance to force. However, attention should also be paid to the fairly large dispersion of the results, which may indicate a heterogeneity of the structure between the samples.

Similar results were obtained by Bernal et al. in their research on geopolymeric composites reinforced with steel fibers. Mechanical tests showed a decrease in compressive strength by about 25%, even the increase in fiber content from 40 kg/m^3^ to 120 kg/m^3^ did not affect the compressive strength. However, they noted better properties in flexural tests and pointed an increase in strength by 70% compared to the base material [51]. A significant increase was seen in the flexural strength—the amount of steel fibers enhanced the flexural behavior was developed. The flexural strength was 75% higher with the addition of 0.5% and almost twice as high with the addition of 1.0% by weight. The addition of melamine fibers in the amount of 0.5% by weight increased the bending strength by about 50%. Introduction higher amount of melamine fibers up to 1% by weight also promoted better flexural properties. However, the increase was already lower, and reached about 20% rise compared to the unmodified material.

Because of a lack of investigations for melamine fibers as a reinforcement for geopolymer composites, the achieved results are discussed with other polymer fibers used as a reinforcement in geopolymer matrix. Puertas et al. and Zhang et al. conducted research on geopolymer composites with polypropylene fibers. The fiber content was 0.5 and 1.0%. The research showed that the addition of polypropylene fibers improved the compressive strength in the initial phase, i.e., after three days of manufacturing, and then the compressive strength declined. Interestingly, they obtained different results by analyzing the bending properties. Zhang et al. noticed that the flexural strength was almost doubled, while Puertas et al. reported a decrease in flexural strength after introduction polypropylene fibers [52,53]. The diversity of the research results is most likely related to the poor wettability of polymer fibers [54].

The last stage of research involved the preparation and examination of geopolymer composites reinforced with hybrid combination of steel and melamine fibers. The proportions of the fibers were changed on the basis of replacement, in this way melamine fibers were replaced with steel fibers every 0.2% of content until the melamine fibers were completely replaced. The maximum content of the fibers was 1.0% by weight.

**Figure 6:**
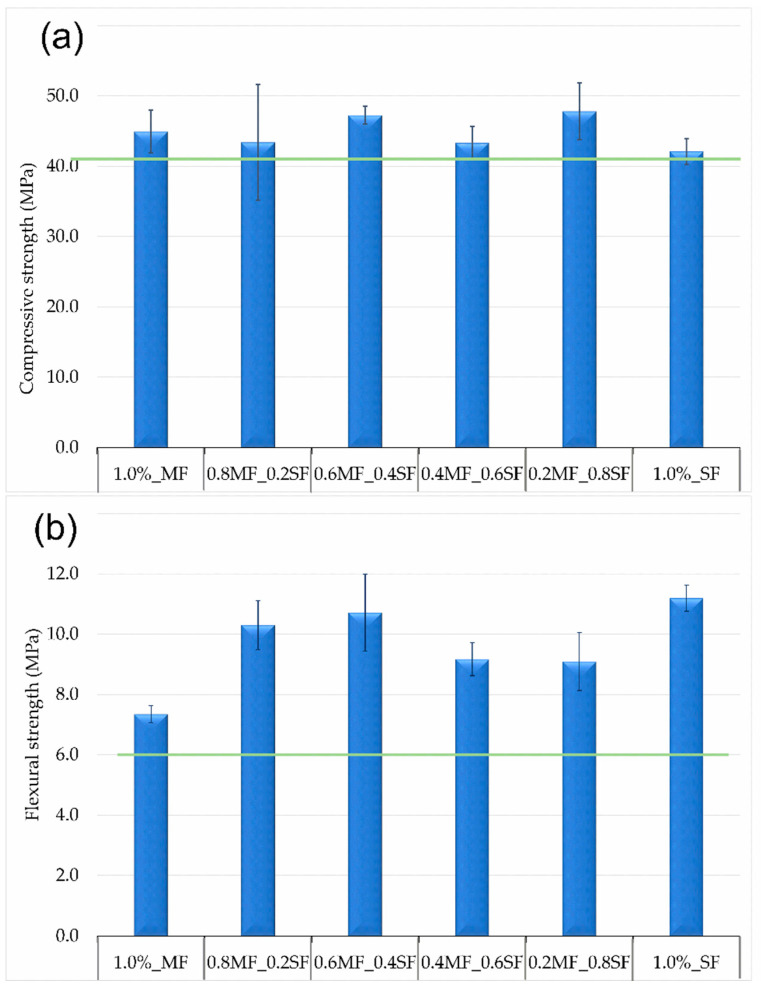
Compressive 6 (**a**) and flexural strength 6 (**b**) of hybrid type reinforcement composites.

Figure 6a,b presents the results obtained from the bending and compression tests. The density of the tested composites (Figure 4) increased with the increase of the steel fibers content, reached the value of 1.85 g/cm^3^. The density of the composite with melamine fiber was 1.75 g/cm^3^. Figure 6b displays changes in flexural strength. Initially, as the amount of steel fiber increased, the bending strength also increased, achieving the best results with 0.6% by weight of melamine fiber and 0.4% of steel fiber, while further enhancement of the steel fiber content caused a decrease in bending strength.

Analyzing the compressive strength, the synergistic effect of melamine fibers with steel fibers can be noticed. The composite containing 1.0% by weight of steel fiber was characterized by a compressive strength of 42.1 MPa, while the composite containing the same content of melamine fibers had a compressive strength on the level of 44.9 MPa. Research on hybrid materials revelated that the combination of both types of fibers but not exceeding the maximum value of 1% by weight, has the highest compressive strength, obtaining values of 47.2 MPa (0.6MF_0.4SF) and 47.8 MPa (0.3MF_0.8SF). This is most likely related to the best fiber content in the amount of 0.5% by weight, analyzing previous results, in which the composite containing 0.5% by weight of steel fibers showed a compressive strength of 46.4 MPa, and a melamine fiber strength of 53.1 MPa.

Sukontasukkul et al. presented a similar study in which the mechanical behavior of hybrid composites reinforced with steel and polypropylene fibers was analyzed. Single type of fiber caused an increase in bending strength and compressive strength. It was also found that the increase in mechanical resistance is related to the effect of bridging the fibers by cracks. Additionally, they showed that the hybrid system had a positive effect on the properties of the composite. However, flexural strength had been shown to improve with the percentage enhancement of steel fibers in the mixture. It was also found that the fiber replacement system appears to perform better than addition fibers system. Introduction more than 1% by volume of reinforcement worsened the previously obtained results. High fiber content made mixing difficult, resulting in the blend thickening, thus affecting the uneven distribution of fibers and increasing the porosity [3].

### 3.3. Influence on Mechanical Properties of Geopolymers the Hybrid Reinforcement

On the basis of the presented research results, the following assumptions can be made:

- Each of the introduced additives increases the mechanical properties of investigated geopolymer composites in relation to the unreinforced material.
- Melamine fibers provide greater resistance to compressive load, and steel fibers are more effective in resisting bending stress.
- The best results were obtained with the content of fibers not exceeding 0.5% by weight.
- As the content of steel fibers increases from 0.5 to 1.0% by weight, the bending strength increases, but the same increase of melamine fibers causes decreases in flexural resistance. However, the value of the flexural strength is still higher than that for unmodified geopolymer;
- Hybrid combination of steel fibers with melamine fibers in the total amount of 1% by weight gives better results in compressive strength compared to composites containing the same amount of fibers but with a single type of reinforcement.

The properties of geopolymer composites depend on many factors, which include the mixing process, workability, shrinkage during drying, density, type of reinforcement, and many others. Melamine fibers are multifilament fibers. Research on polypropylene fibers, which are also multifilament additives, showed that the fibers during dry mixing do not separate and do not disperse in the matrix of the material causing large differences in the density and the structure of the composite because the binder is not able to penetrate the fiber network. A good solution may be to mix the fibers with an alkaline activator solution first to break the fiber bundles and then mix with dry aluminosilicates and other fillers. This may improve the fiber wetting process and, as a consequence, results in a greater fiber–matrix interaction [50].

The content of the fibers is also extremely important. The research presented in this paper suggests that the addition of polymer fibers should not exceed 0.5%, as a further increase in the amount of fibers worsens the compression properties.

An increase in the amount of the fibers above a certain limit leads to uneven dispersion and the formation of lumps or balls. Moreover, even a very fluid matrix material may not pass properly through the overloaded network of fibers, therefore, despite the excellent mechanical properties, the fibers reduce the workability of the mass, which causes the excessive formation of voids and insufficient compaction [50].

The properties of geopolymer composites during bending stress are dependent on the type of fibers and the matrix-fiber interaction. The onset of cracking occurs when the bending stress exceeds the bending capacity of the geopolymer matrix and increases until it encounters the fiber reinforcement, and then propagates based on the fiber-matrix interaction. Lower resistance to bending for composites with melamine fiber are related to weak adhesion and poor wettability of polymer fibers. Similarly, polymer fibers, due to their chemical inertness and low surface energy, result in poor bonding to the matrix and lower resistance to bending stresses. A higher content of bridging fibers, over a longer length, can significantly affect the amount of energy absorption by the composite and thus enhance the mechanism of resistance to crack growth due to entanglement of the fibers, but this mechanism is not always sufficient. Steel fibers have a different nature. Their hydrophilic surface causes a strong contact between the fibers and the geopolymer matrix; furthermore, the shrinkage process over time increases the clamping pressure and strengthens the interphase between the components. Consequently, it provides higher bending strength and energy absorption [32].

The behavior of geopolymers under compression is highly related to the brittleness, pore structure and microcrack distribution. When the geopolymeric material is subjected to uniaxial compression, axial microcracks appear that split parallel to the direction of compression. It is correlated to the concentration of transverse tensile stresses in front of the formed crack, which causes their growth in the direction of the compressive load. The presence of fibers reduces the initiation and propagation of cracks. It is connected with the necessity to provide more energy needed to pull the fibers out of the matrix so that the propagation of the crack can be continued. However, irrespective of the type of fiber, an increase in compressive strength is more expected with a fiber content below 2%, while above this value, the fiber may be adversely affected. This can be ascribed to the significant increase in porosity above the critical fiber concentration, which is often between 0.2% and 2% [50].

## 4. Conclusions

The paper presents geopolymer composites reinforced with steel and melamine fibers, as well as their hybrid combination. Melamine fibers can be successfully used to increase resistance to axial compression, while steel fibers have a better ability to dissipate stress during three-point bending. The presented test results proved that each of the additives improves the bending and compression properties in relation to the unreinforced base material. It was found that a single type of reinforcement provides the highest values of compressive strength when the amount of fiber does not exceed 0.5% by weight. A hybrid reinforcement system of steel and melamine fibers can ensure a synergistic effect of strengthening. Hybrid composites containing the maximum 1% by weight of steel and melamine fibers were characterized by better compressive strength results compared to the composites with a single type of fiber density in the amount of 1% by weight.

## Figures and Tables

**Table 1:** Examples of applied fibers [4].

Type of Fiber	Name of the Fiber	Density (g/cm^3^)	Tensile Modulus * (GPa)	Price ** (Euro/kg)	Aim of Addition
Natural	Cotton [5,6]	1.5–1.6	5–17	≈2	Increases compressive and flexural strength, stiffness and resistance to brittle cracking
	Banana [7]	1.35	3.48	≈1.5	Increases compressive and flexural strength
	Sisal [8]	1.5	10–30	≈1.8	Increase compressive strength, and bending strength
	Lien [9]	1.5	50–70	≈35	
	Raffia [10]		28–36	≈5–15	
	Juta [11]	1.4–1.5	20–30	≈35	
	Basalt [12,13]	2.7	88–92	≈5	Increases the compressive strength, and the modulus of rupture of the concrete.
	Coconut [14]	1.1–1.3	4–15	≈35	Increases compressive, and bending strength
Organic	Polyethylene [15]	0.96	5–30	≈4	Shrinkage and thermal crack control, corrosive resistant; Increase flexural and tensile strength; Increase impact and abrasion resistance; Material weight reduction.
	Polypropylene [15,16]	0.91	5–10	≈5	
	Polyvinyl alcohol [17]	1.3	25–40	≈15	Improve impact, shatter and abrasion resistance of concrete; Enhances durability and toughness of concrete; Material weight reduction.
	Polyamide [18]	1.45	50–120	≈35–85	
Inorganic	Steel fiber [19]	7.8	210	≈1	Increases tensile strength; Bolster impact and abrasion resistance; Hooked-end configuration enables prediction of failure point; Increases post-crack flexural strength
	Glass fiber [20]	2.7	70	≈10	Reduces the formation of plastic shrinkage cracking in concrete; Improves impact resistance of concrete; Enhances durability and toughness of concrete.
	Carbon fiber [5,21]	1.6–1.8	230	≈25	Increases in mechanical strength and tensile modulus; Corrosion reduction

* the given data depend on the manufacturer of the individual fibres. ** the prices given are the prices of individual producers and may vary depending on the supplier.

**Table 2:** Description of the fibers.

Material	Shape of Fiber	Length (mm)	Cross-Sectional Dimensions of Fiber (mm)	Tensile Strength of Fiber (MPa)	Tensile Modulus (GPa)
Steel fiber (SF)	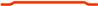 hooked-ended, flattened cross-section	51 ± 0.2	width: 0.9 ± 0.05 thickness: 1.1 ± 0.3	1190	210
Melamine fiber (MF)	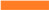 dimension circa 7 µm *	≈5	Rectangular width: 3.6 mm length: 0.8 mm	248	6.9

* Diameter of single melamine fiber which created a bundle of fibers.

**Table 3:** Designation of manufactured composites.

Type	Designation	Mixture Proportion (% by Weight/by Volume)				NaOH Solution
		Fly Ash	Sand	Steel Fiber	Melamine Fiber	
Single type	Plain Geopolymer	50	50	-	-	10 M sodium hydroxide solution + water glass (1200 mL in total)
	0.5 SF	49.75	49.75	0.5/3.9	-	
	1.0 SF	49.5	49.5	1.0/7.9	-	
	0.5 MF	49.75	49.75	-	0.5/6.4	
	1.0 MF	49.5	49.5	-	1.0/12.6	
Hybrid fiber	1.0 MF (repeat)	49.5	49.5	-	1.0/3.4	
	0.8MF_0.2SF	49.5	49.5	0.2/1.6	0.8/10.2	
	0.6MF_0.4SF	49.5	49.5	0.4/3.1	0.6/7.7	
	0.4MF_0.6SF	49.5	49.5	0.6/4.7	0.4/5.1	
	0.2MF_0.8SF	49.5	49.5	0.8/6.2	0.2/2.6	
	1.0 SF (repeat)	49.5	49.5	1.0/7.9	-	

**Table 4:** Identified phases with their percentage share.

**Precursor**	**Identified Phase**		**Percentages Content (%)**
Fly Ash	**Name**	**Chemical Formula**	
	Quartz	SiO_2_	42.3
	Mullite	Al_6_Si_2_O_13_	54.8
	Hematite	Fe_2_O_3_	0.6
	Magnetite	Fe_3_O_4_	0.5
	Anhydrite	CaSO_4_	1.4
	Rutile	TiO_2_	0.4
